# Genetic Characterization of Soybean Rhizobia Isolated from Different Ecological Zones in North-Eastern Afghanistan

**DOI:** 10.1264/jsme2.ME16119

**Published:** 2017-03-17

**Authors:** Safiullah Habibi, Abdul ghani Ayubi, Naoko Ohkama-Ohtsu, Hitoshi Sekimoto, Tadashi Yokoyama

**Affiliations:** 1 United Graduate School of Agriculture, Tokyo University of Agriculture and TechnologyJapan; 2 Faculty of Agriculture, Kabul UniversityAfghanistan; 3 Institute of Agriculture, Tokyo University of Agriculture and TechnologyJapan; 4 Faculty of Agriculture, Utsunomiya UniversityJapan

**Keywords:** Afghanistan, *Glycine max*, *Bradyrhizobium*, *Ensifer*, Symbiotic genes

## Abstract

Seventy rhizobial isolates were obtained from the root nodules of two soybean (*Glycine max*) cultivars: Japanese cultivar Enrei and USA cultivar Stine3300, which were inoculated with different soil samples from Afghanistan. In order to study the genetic properties of the isolates, the DNA sequences of the 16S rRNA gene and symbiotic genes (*nodD1* and *nifD*) were elucidated. Furthermore, the isolates were inoculated into the roots of two soybean cultivars, and root nodule numbers and nitrogen fixation abilities were subsequently evaluated in order to assess symbiotic performance. Based on 16S rRNA gene sequences, the Afghanistan isolates obtained from soybean root nodules were classified into two genera, *Bradyrhizobium* and *Ensifer*. *Bradyrhizobium* isolates accounted for 54.3% (38) of the isolates, and these isolates had a close relationship with *Bradyrhizobium liaoningense* and *B. yuanmingense*. Five out of the 38 *Bradyrhizobium* isolates showed a novel lineage for *B. liaoningense* and *B. yuanmingense*. Thirty-two out of the 70 isolates were identified as *Ensifer fredii*. An *Ensifer* isolate had identical *nodD1* and *nifD* sequences to those in *B. yuanmingense*. This result indicated that the horizontal gene transfer of symbiotic genes occurred from *Bradyrhizobium* to *Ensifer* in Afghanistan soil. The symbiotic performance of the 14 tested isolates from the root nodules of the two soybean cultivars indicated that *Bradyrhizobium* isolates exhibited stronger acetylene reduction activities than *Ensifer* isolates. This is the first study to genetically characterize soybean-nodulating rhizobia in Afghanistan soil.

Soybean (*Glycine max* (L.) Merr.) originated in eastern Asia, and is now being cultivated worldwide under various climatic conditions. Soybean has significant agronomic and nutritional importance because of the high concentrations of protein and oil in its grains. In order to achieve optimum growth and a high yield, soybean has strong demands for nitrogen (N) to synthesize protein. However, nitrogen is one of the nutrients that most frequently limits plant growth in many soils. Soybean may obtain a large part of its nitrogen requirement by establishing N_2_-fixing symbiosis with rhizobia. Soybean-nodulating rhizobia have the ability to nodulate and establish effective symbiosis with soybean plants such as other legumes. They have been isolated and genetically characterized in different continents and climatic zones. These bacteria are Gram-negative and genetically diverse, and have been classified into different genera and species. Soybean is nodulated by fast- and slow-growing rhizobia. The major slow-growing soybean-nodulating bradyrhizobia are *Bradyrhizobium japonicum* (19), *B. elkanii* (23), *B. liaoningense* (45), and *B. yuanmingense* (3, 31), while the fast-growing rhizobia are *Ensifer fredii* (also known as *Sinorhizobium fredii*) (10, 28), *Mesorhizobium albiziae* (44), *M. septentrionale*, *M. temperatum* (15), and *M. tianshanense* (9). The genetic diversity and geographical distribution of soybean rhizobia are related to numerous factors such as pH (47) and climate (1). Among three well-known soybean symbionts (*B. japonicum*, *B. elkanii*, and *B. liaoningense*), *B. liaoningense* showed more biogeographic specificity (1) than *B. japonicum* and *B. elkanii*. However, *B. liaoningense* has not yet been surveyed worldwide. *B. liaoningense* is an extra slow-growing species and has been isolated from different geographical regions in Asia (1–3, 8, 17, 25, 34, 43, 45–47). *Ensifer* species are fast-growing rhizobia that were initially isolated in China (20), and then identified in saline-alkaline soils in a large number of studies (3, 4, 17, 23, 25, 26, 34).

Afghanistan is a landlocked country located in the center of Asia and forms part of South Asia, Central Asia, and Greater Middle Eastern Asia. It is bordered by Pakistan in the south and east, Iran in the west, Turkmenistan, Uzbekistan, and Tajikistan in the north, and China in the far northeast, as shown in Fig. 1.

Agriculture is an important sector in Afghanistan, and its economy accounts for approximately one third of the gross domestic product (GDP) (20). Agriculture production is regarded as the key component of the economy and livelihood (20). Soybean cultivation was launched in 1881 (36), and followed with the last yield of 2.5 t ha^−1^ (18). The Afghanistan government and American NGOs in Afghanistan recently introduced new soybean varieties including Stine3300. In the present study, we attempted to evaluate the host specificities of Asian (Enrei) and American (Stine3300) soybean varieties with Afghanistan soybean microsymbionts. Six soil samples were collected from various fields in different agricultural-ecological-climatic zones, including the main cropping area. Samples were used as inoculants for two soybean cultivars, the Asiatic cultivar (Japanese c.v. Enrei) and USA cultivar (Stine3300), in order to isolate the root nodule bacteria associated with soybean. Rhizobial diversity was evaluated using several molecular approaches, including the 16S rRNA gene and symbiotic genes (*nifD* and *nodD1*). The most promising isolates based on their growth performance in inoculated soybean cultivars were selected as candidates to develop bio-fertilizers for soybean in Afghanistan.

## Materials and Methods

### Soil sampling

**Fig. 1:**
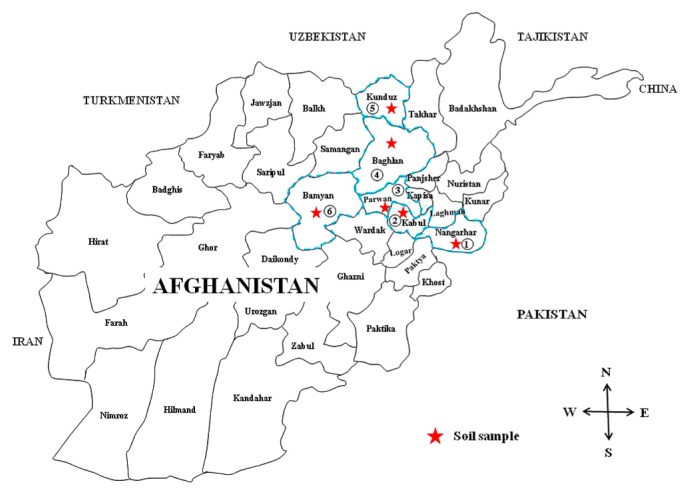
Map of Afghanistan showing soil sample collection sites

In order to isolate soybean-nodulating rhizobia, 6 soil samples were collected from various legumes fields (alfalfa, mung bean, and soybean) in different agricultural climatic regions at depths of 0 to 20 cm (Fig. 1, Table 1). Soil sampling was performed between August 15th, 2012 and September 10th, 2012. These samples were transported by air to Japan under strict control by the Yokohama Plant Quarantine office. After arriving at Narita in Japan, these soil samples were maintained at 4°C in a cold room. Rhizobium isolation using the samples imported from Afghanistan was conducted between October and November, 2012. The sampled sites of the soils had no history of soybean cultivation, except for Kabul (recently started soybean cultivation), and no bacterial inoculation. Therefore, the isolated strains were considered to be indigenous to Afghanistan.

### Isolation of indigenous rhizobia

The seeds of two soybean cultivars: c.v. Stine3300 (USA variety) and c.v. Enrei (Japanese variety), were surface-sterilized by immersion in 70% ethanol for 30 s and then in 3% sodium hypochlorite solution for 3 min before being exhaustively washed with sterile water. Regarding germination, sterilized seeds were incubated on 0.9% agar medium at 25°C for 1.5 d. Five-fold dilutions (1 g 5 mL^−1^) of soil suspensions were used as inoculants. Each inoculant was applied to 300-mL glass jars (except the control) containing germinated seeds and sterilized vermiculite. The jars were transferred to a growth chamber and kept under controlled conditions (16-h light/8-h dark photoperiod, at 25°C/18°C day/night temperatures). Plant growth was supported by adding sterilized nitrogen-free nutrient solution (7) to the jar up to the 60% soil moisture level. After four weeks, whole plants were removed from the jars, washed in running tap water to remove vermiculite, and the root nodules were harvested. Root nodules were surface-sterilized by immersion in 70% ethanol for 30 s and in 3% sodium hypochlorite for 3 min, and then washed five times with sterile water. Each nodule was crushed in 100–200 μL glycerol solution [15% (v/v)] to obtain a turbid suspension. An aliquot (10 μL) of the suspension was streaked onto yeast extract mannitol agar (YEM) (37) plates and incubated at 28°C for 3–7 d. The remaining suspension was maintained at −80°C. Single colonies were picked and checked for purity by repeated streaking onto fresh YEM. Isolates were transferred into 15% glycerol stocks and stored at −80°C for long-term maintenance and for short-term maintenance were transferred into slant stocks and stored at 4°C.

### DNA extraction

Isolates were grown in 20 ml YEM broth medium at 25°C for 4–7 d. Prior to genomic isolation, cells were harvested and washed twice with equal volumes of TNE buffer (10 mM Tris, 0.1 M NaCl, and 1 mM EDTA, pH 8). Genomic DNA was extracted from isolates using the method described previously by Yokoyama *et al.* (49). The concentration and purity of DNA were checked using a Nano Drop 2000 UV-Vis Spectrophotometer (Thermo Fisher Scientific, Wilmington, DE, USA).

### DNA amplification and sequencing

The PCR amplification and sequencing of DNA fragments of the 16S rRNA, *nifD*, and *nodD1* genes were performed as described by Habibi *et al.* (16). The primer sets used for PCR amplification and sequencing are shown in Table S1. PCR products were sequenced using an ABI PRISM 3500 genetic analyzer (Applied Biosystems) according to the manufacturer’s protocols. DNA sequences were compared to the GenBank nucleotide sequence database using BLAST (NCBI). Sequence alignment and construction of the phylogenetic tree were performed using MEGA version 6.06 (41).

### Nucleotide sequence accession numbers

The 16S rRNA, *nodD1*, and *nifD* sequences have been deposited in the DNA Data bank of Japan under the accession numbers AB901294–AB901363 for 16S rRNA, LC009304–LC009373 for *nodD1*, and AB982147–AB982216 for *nifD*.

### Symbiotic performance

The same soybean cultivars (Enrei and Stine3300) were used to confirm compatibility for effective nodule formation by each isolate. Fourteen isolates were selected from different genotypes as representatives (Table 2) and grown in 15 mL YEM broth at 28°C for 3–7 d. In order to evaluate the cell numbers of the inoculants, bacterial cells were collected by centrifugation at 28,000×*g* at 4°C for 10 min, and then washed twice with 1× TNE solution. Colony-forming units (CFU) were counted using the dilution plate count method. Inoculant cells (1–1.5 mL) at a density of 10^8^ CFU were applied to a seed in a growing jar. The jars were transferred to a growth chamber and kept under controlled conditions (16-h light/8-h dark photoperiod, at 25°C/18°C day/night temperatures). After four weeks, whole plants were removed from the jars, washed in running tap water to remove vermiculite, and the root nodules were harvested. This experiment was performed in a completely randomized block design that consisted of three replicates for each treatment. Different plant growth parameters such as shoot weight (fresh and dry), root weight (fresh and dry), root nodule weight (fresh and dry), and nodule numbers were assessed. Plant shoots and root nodules were dried at 80°C for 48 h in order to obtain dry weights. In the acetylene reduction assay, fresh roots that contained root nodules were placed in a 300-mL jar, the air in the jar was supplemented with 10% acetylene (v/v) for each treatment, and the jar was incubated at room temperature (25°C) for 1 h. The concentration of ethylene in the jar was measured using a gas chromatograph (Shimadzu 2014AF, Kyoto, Japan). *B. japonicum* USDA 110 (31) was included as a positive control in the symbiotic test. The significance of differences between inoculated and un-inoculated plants was assessed using Tukey’s test (*P*<0.05) (Table 2).

## Results

### Root nodule numbers of Enrei and Stine3300 in combination with 6 different soils

Eighty nodules were harvested from two soybean cultivars (29 from Enrei and 51 from Stine3300). Two soil samples (Kabul and Bamyan) failed to form root nodules in either soybean cultivar (Table 1). We also did not observe any root nodules in soybean plants growing in Kabul Province at the time of soil sampling. No root nodule was found in the Enrei cultivar after inoculations with five of the soil samples (samples 2, 3, 4, 5, and 6 in Table 1). The only soil sample that produced a large number of root nodules in both cultivars was from Nangarhar (sample 1 in Table 1). The Stine3300 cultivar showed higher adaptability than the Enrei cultivar to the indigenous soybean rhizobia in three other soil samples (samples 3, 4, and 5 in Table 1). Seventy rhizobial isolates were obtained from the nodules (Table 3).

### Phylogenetic analysis based on the 16S rRNA gene

**Fig. 2:**
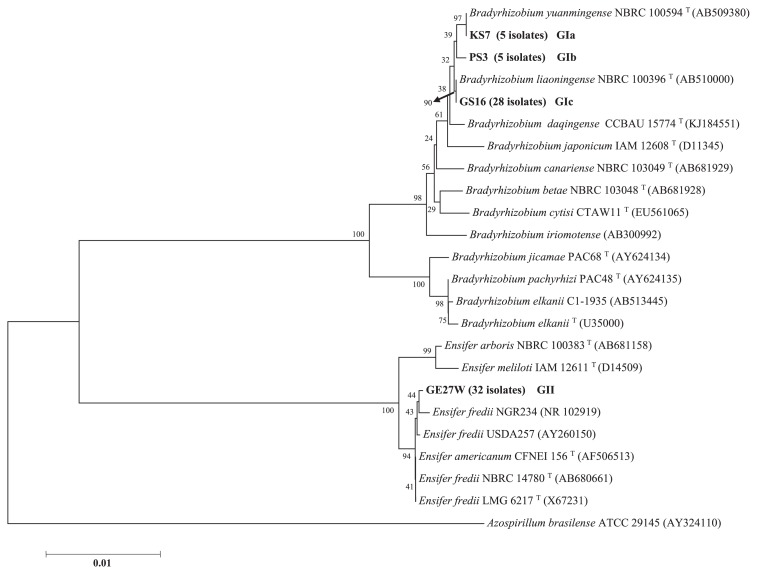
Phylogenetic tree constructed using partial 16S rRNA nucleotide sequences (1331 bp) from 70 Afghanistan isolates. The type strains of the species that belong to the *Bradyrhizobium* and *Ensifer* genera are shown. GenBank accession numbers are given in brackets. The numbers at the nodes indicate the level of bootstrap support based on a neighbor-joining analysis. *Azospirillum brasilense* ATCC 29145 was used as the outgroup.

The almost full length of the 16S rRNA gene (1331 bp) of 70 isolates was sequenced and their accession numbers (AB901294 to AB901363) are shown in Table 3. These isolates were divided phylogenetically into two groups (GI and GII) based on the 16S rRNA sequence analysis, as shown in Fig. 2. The GI group contained 38 isolates (54.3% of the total), while the GII group included 32 isolates (45.7%). The GI phylogenetic group was further divided into three subgroups: GIa, which contained five isolates and showed a close relationship (99%) to *B. yuanmingense* NBRC 100594 (reference strain); GIb, which included five isolates and separated from *B. yuanmingense* and *B. liaoningense*; and GIc, which included 28 isolates and showed high similarity (100%) to *B. liaoningense* NBRC 100396 (reference strain). The reference strains of the remaining *Bradyrhizobium* species were genetically distant from the Afghanistan isolates in the phylogenetic tree, as shown in Fig. 2. GII group isolates were mainly obtained from samples of Nangarhar Province, which has a hot desert climate, and showed maximum similarity (99%) to *E. fredii* NGR234 followed by *E. americanum* CFNEI 156, while *E. arboris* NBRC 100383 and *E. meliloti* IAM 12611 formed a separate cluster from the Afghanistan isolates.

### Phylogenetic analysis based on the *nod D1* gene

**Fig. 3:**
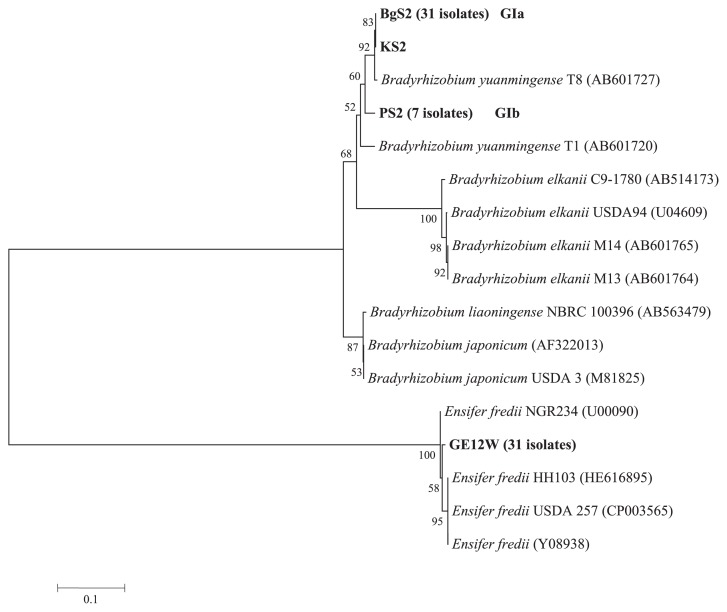
Phylogenetic tree constructed using partial nucleotide *nodD1*sequences (658 bp) from 70 Afghanistan isolates. GenBank accession numbers are given in brackets. The numbers at the nodes indicate the level of bootstrap support based on a neighbor-joining analysis. One isolate (KS2) from a *Ensifer* species showed close similarities to *Bradyrhizobium yuanmingense* and was categorized into the GIa subgroup.

Based on differences in the *nodD1* (658 bp) phylogenetic analysis, 70 isolates were divided into two major groups, as shown in Fig. 3 and Table 3. The GI group contained 39 isolates (55.7% of the total), while the GII group contained 31 isolates (44.3%). The GI group was divided into two subgroups: GIa, which contained 32 isolates and had a close relationship (98%) with *B. yuanmingense*; and GIb, which contained seven isolates and separated from *B. yuanmingense* T8 AB601727 and *B. yuanmingense* T1 (AB601720). All *B. liaoningense* isolated from Afghanistan soil shown in Fig. 2 had the *nod* genes of *B. yuanmingense*. The GII group had a close relationship (99%) with *E. fredii* and reference strains. Interestingly, one isolate (KS2) that was classified into the *Ensifer* group based on 16S rRNA sequences showed a close relationship (99%) with *B. yuanmingense* based on *nodD1* sequences (Table 3).

### Phylogenetic analysis based on the *nifD* gene

**Fig. 4:**
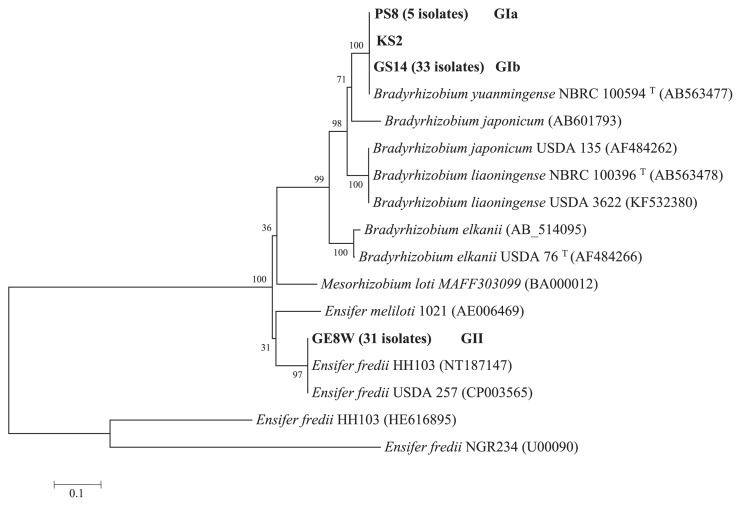
Phylogenetic trees constructed using partial *nifD* nucleotide sequences (677 bp) from 70 Afghanistan isolates. GenBank accession numbers are given in brackets. The numbers at the nodes indicate the level of bootstrap support based on a neighbor-joining analysis. The KS2 isolate was included in the GIb subgroup and showed close similarities to *Bradyrhizobium yuanmingense* NBRC 100594.

Seventy tested isolates formed two groups in the phylogenetic tree based on *nifD* sequences (677 bp), as shown in Fig. 4 and Table 3. The GI group contained 39 isolates (55.7% of the total), while the GII group contained 31 isolates (44.3%), as shown in Table 3. The GI group showed a close relationship (99%) with *B. yuanmingense* and was divided into two subgroups: GIa, which contained 34 isolates, and GIb, which contained five isolates. In the GIb subgroup, four isolates (PS2, PS5W, PS6, and PS8) were from the same region (Parwan Province), while the KS3 isolate was from Kunduz Province. All isolates in GIa and GIb belonged to *Bradyrhizobium* and had close relationships with the *B. yuanmingense* isolates. Furthermore, all *B. liaoningense* isolated in Afghanistan soil shown in Table 3 and Fig. 2 had the *nif* genes of *B. yuanmingense*. The KS2 isolate belonged to the *Ensifer* group based on 16S rRNA sequences and was classified into the GIb subgroup based on *nifD* sequences. The GII group showed a close relationship (99%) with *E. fredii* USDA 257 and *E. fredii* HH103.

### Symbiotic performances

The symbiotic performances of the isolates belonging to different phylogenetic groups based on the 16S rRNA, *nifD*, and *nodD1*gene sequences are shown in Table 2. Fourteen representative isolates produced root nodules when inoculated onto the seeds of the Stine3300 and Enrei cultivars. The root nodules that developed in soybean by the *Bradyrhizobium* isolates exhibited slightly stronger acetylene reduction activities (ARA) than the *Ensifer* isolates. However, no significant differences were observed in root nodule numbers in the two soybean cultivars among the *Bradyrhizobium* and *Ensifer* isolates.

Isolates in the GI group defined by the *nodD1* sequence analysis belonged to the genus *Bradyrhizobium*, and KS3 and GE3 showed high root nodule numbers in both soybean cultivars. Of the eight isolates tested, the root nodule numbers of six (KS3, BgS4, PS3, GE3, KS12, and PS8) were higher in Enrei than in Stine3300. In the ARA, the PS2 and KS12 isolates, which belonged to the GIa and GIb groups, respectively, based on the phylogenetic tree constructed using the *nifD* sequences, exhibited strong ARA activities in the Stine3300 cultivar. In the root nodules of Enrei, GE3 and KS12 also exhibited strong ARA activities, with KS12 exhibiting particularly strong ARA activity in both cultivars. GE3 showed a higher biomass amount with the strongest ARA activity in Enrei, while KS3 increased the biomass of Enrei and had the highest root nodule number among all the isolates tested. No significant differences were observed in symbiotic performances among the three GI subgroups of *Bradyrhizobium* isolates. In the GII group based on the phylogenetic tree constructed using the *nodD1* sequences, the isolates were *E. fredii* and generally showed higher numbers of root nodules in Enrei than in Stine3300 as well as stronger ARA activities (excluding the GE24W isolate). GE6W showed high nodulation adaptability in the two cultivars. In the GII group based on the phylogenetic tree constructed using the *nifD* sequences, the isolates in the root nodules obtained from Enrei exhibited strong ARA activities, with the exception of GE24W. GS4 showed relatively good symbiotic performance based on the high root nodule number and strong ARA activity. GE6W isolates showed high biomass amounts in the two cultivars, with high root nodule numbers; however, ARA values were not very high. The KS2 isolate was grouped with *Bradyrhizobium* based on the trees constructed using the *nifD* and *nodD1* sequences (Table 2, Part B). The symbiotic performance of KS2 was similar to that of GE24W, which belonged to the *Ensifer* type.

## Discussion

### Isolation of root nodule bacteria

Six soil samples were collected from different sites (North to South-eastern) in Afghanistan (Fig. 1), inoculated into two soybean cultivars (Enrei and Stine3300) as trap hosts, and rhizobia were isolated from their root nodules. A soil sample collected in the Nangarhar Province showed a high frequency for the distribution of soybean rhizobia; this may be because of continuous mung bean cultivation in this region (4, 51). In contrast, two soil samples obtained from Kabul and Bamyan provinces showed no distribution of soybean rhizobia. In the soil sample from Kabul Province, we were unable to confirm any soybean rhizobia despite soybean having been cultivated in this region. This result was consistent with our observation that no root nodule was observed in the soybean plants cultivated in Japan. The Japanese Enrei cultivar had a low level of host specificity; among the six soils tested, only the soil sample from Nangarhar province contained soybean rhizobia with the ability to nodulate with cv. Enrei.

### Soybean rhizobia communities in Afghanistan soils

Based on 16S rRNA gene sequences, 70 Afghanistan isolates were categorized into two major clusters, the genus *Bradyrhizobium* and genus *Ensifer* (Fig. 2). *Bradyrhizobium* isolates were further divided into three subgroups: GIa, GIb, and GIc. GIa isolates shared almost identical 16S rRNA gene sequences with that of *B. yuanmingense* NBRC 100594^T^, while GIb isolates were similar to, but also different from *B. yuanmingense* NBRC 100594^T^. *B. yuanmingense* was originally described by Yao *et al.* (48) as a microsymbiont of *Lespedeza* sp.. Host-specificity studies reported ineffective root nodule formation in soybean by *B. yuanmingense* isolates (46, 48); however, the effective nodulation capability of *B. yuanmingense* was recently demonstrated (3, 31). The GIc group contained 28 isolates and had a close relationship with *B. liaoningense*, which was firstly reported in China (44) and then frequently described in other studies (2, 3, 17, 25, 34, 43, 46, 47). Li *et al.* (25) reported that *B. yuanmingense* and *B. liaoningense* were the predominant soybean microsymbionts in alkaline and saline soils. The GII group contained *Ensifer* isolates, fast-growing bacteria isolated from the root nodules of *Lablab purpureus* in Papua New Guinea alkaline soil (30). *E.* (*Sinorhizobium*) *fredii* was initially isolated from an old Peking soybean variety (*G. max*) in China (21, 22), and their effective nitrogen-fixing symbioses with Asian soybean cultivars (*G. max* and *G. soja*) have been reported (12, 21, 38). Subsequent studies showed that *E. fredii* also established effective nodules with some American soybean cultivars (5). *Ensifer* species were initially included in the genus *Rhizobium* and classified as *Rhizobium fredii* (35), they were then assigned as *S. fredii* by Chen *et al.* (10), and finally reassigned to the genus *Ensifer* (Young 2003). *Ensifer* species are acid-producing rhizobia (10), and are well adapted to saline-alkaline soils (3, 17, 25, 26, 28, 34, 40); their ability to produce acid substances may contribute to the survival of rhizobia under alkaline conditions (33). Regarding *E. fredii* isolated from soil samples from Nangarhar and Kunduz provinces, soil pH were 7.66 and 7.65, respectively. *E. fredii* NGR234 has a wide host range in leguminous plants (112 genera), in which it may form either determinate or indeterminate nodules (30), as well as in the non-legume tropical tree *Parasponia andersonii* (24). *E. fredii* NGR234 has a phylogenetically close relationship with *E. fredii* USDA257, and both strains share most of their genomic backgrounds (29).

### Symbiotic gene diversity of Afghanistan isolates

Among 38 *Bradyrhizobium* isolates from Afghanistan, 28 had a close relationship with *B. liaoningense* based on 16S rRNA gene sequences, as shown in Table 3 and Fig. 2. All 28 isolates belonging to *B. liaoningense* had the *nodD1* and *nifD* genes of *B. yuanmingense*. Regarding the distribution of *B. yuanmingense*, Risal *et al.* (31, 32) reported that *B. yuanmingense* was the predominant soybean- and mung bean-infecting bradyrhizobia in Nepal. Appunu *et al.* (3) also showed that *B. yuanmingense* was the predominant soybean-bradyrhizobia in India. Although the distribution of *B. yuanmingense* was not clear in Pakistan, *Bradyrhizobium* harboring the *nod* and *nif* genes of *B. yuanmingense* may be the most adaptable symbiotic rhizobia in the western parts of Asia.

Based on the phylogenetic analysis of the *nifD* and *nodD1* genes, 31 *Ensifer* isolates were grouped with *E. fredii* HH103, *E. fredii* USDA 257, and *E. fredii* NGR234. Interestingly, the *nifD* and *nodD1* regions of one isolate categorized into *Ensifer* had close relationships with the corresponding regions in *B. yuanmingense* (Figs. 3 and 4, and Table 2), implying that symbiotic gene transfer from *B. yuanmingense* to *E. fredii* occurred in Kunduz soil, as described by Sullivan *et al.* (39) for *Lotus*-nodulating rhizobia. Horizontal or lateral gene transfer has long been known to occur among prokaryotes (11), in which it plays a major role in prokaryote genome evolution (14, 27). Barcellos *et al.* (6) and Djedidi *et al*. (13) also recently described horizontal gene transfer among different genera and species. In the present study, we found that the *nodD1* and *nifD* genes of *B. yuanmingense* were transferred to KS2 of *Ensifer* (Tables 2 and 3). However, it currently remains unclear whether the remaining *nod* and *nif* genes in the symbiotic island of *B. yuanmingense* were also transferred to KS2. If all the *nod* and *nif* genes of *B. yuanmingense* were transferred to KS2 and were active, this may explain why the performance of nodulation by the KS2 isolate was similar to those of the isolates of *B. yuanmingense* (Table 2). However, based on the ARA values of the root nodules produced by KS2, the nitrogen fixation performance of KS2 was markedly lower than that of the *B. yuanmingense* nodules, while ARA values were similar to those of *E. fredii*. Further studies are required in order to elucidate the weakness of *nif* gene activities in KS2 isolates.

We found no clear relationship among the ARA values of the *nifD* groups, root nodule numbers in *nodD1* groups, and plant dry weights shown in Table 2. In *Ensifer*, all the isolates tested had the same *nodD* and *nifD* (Table 2, Part A) and belonged to the same phylogenetic group (GII); however, their nodule numbers and ARA values markedly varied. In *Bradyrhizobium*, all the tested isolates exhibited similar tendencies to those of *Ensifer* (Table 2, Part C). This result showed that when effective soybean inoculants for Afghanistan soybean cultivation are obtained, characteristics such as the nodule numbers, ARA values, and plant promotion activities of the target candidates need to be checked.

## Conclusion

Based on the symbiotic performances of the isolates, which were tested with the two soybean cultivars, we propose that the *Ensifer* GS4 and GE6W isolates and *Bradyrhizobium* GE3 isolate are potential candidates to improve soybean cultivation and production in Afghanistan. Soybean is a new legume crop in Afghanistan and its development will contribute greatly to food security in Afghanistan. However, increasing the performance of the soybean crop is a major challenge for Afghanistan farmers, and the efficacy of symbiotic nitrogen fixation may be an important factor for enhancing productivity through the successful management of the soybean and indigenous-rhizobia symbiosis. Our results will contribute to the development of an effective soybean inoculant in Afghanistan. Further studies are required in order to establish a suitable inoculation technology for soybean cultivation in Afghanistan.

## Supplementary Information



## Figures and Tables

**Table 1:** Soil sampling sites and numbers of nodules obtained from two soybean cultivars after their inoculation with soil samples

Soil sample No.	Soil sampling sites	Climate	Latitude and longitude	Previous crop	pH^a^	EC (ds m^−1^)^b^	Number of nodules cultivated from 2 soybean cultivars	

							Enrei	Stine3300
1	Nangarhar	Hot desert climate	34° 25′ N–70° 27′ E	Mung bean	7.66±0.02	0.57±0.01	29	27
2	Kabul	Semi-arid climate	34° 31′ N–69° 11′ E	Soybean	8.10±0.70	2.29±0.16	0	0
3	Parwan	Cold semi-arid climate	35° 07′ N 69° 14′ E	Alfalfa	7.70±0.05	0.61±0.15	0	6
4	Baghlan	Semi-arid climate	36° 08′ N–68° 42′E	Mung bean	7.85±0.05	1.28±0.03	0	4
5	Kunduz	Semi-arid climate	36° 43′ N–68° 52′ E	Mung bean and Maize	7.65±0.06	1.68±0.01	0	14
6	Bamyan	Cold arid and semi-arid	34° 49′ N–67° 49′ E	Alfalfa	8.25±0.06	4.10±0.43	0	0

a Measured with a pH meter in a 1:2.5 (w/v) soil and distilled water solution (42)

b Measured with a conductivity meter in a 1:2.5 (w/v) soil and distilled water solution (42)

**Table 2:** Symbiotic performances of 14 isolates that produced root nodules

Part A: Plant response to the inoculation groups of the *Ensifer* type										

Isolate name	Phylogenetic groups of 16S rRNA	Related species	Phylogenetic groups of *nodD*	Nodule numbers/plant (Stine3300)	Nodule numbers/plant (Enrei)	Phylogenetic groups of *nifD*	ARA (μmol ethylene produced/hr/dry weight of nodule) (Stine3300)	ARA (μmol ethylene produced/hr/dry weight of nodule) (Enrei)	Biomass Stine 3300 (mg plant^−1^)	Biomass Enrei (mg plant^−1^)
GS4	GII	*E. fredii*	GII	54±101)	83.7±23	GII	24.2±8.99	48.36±30.182)	1978.7±180*1)	2991.4±307*2)
GS1	GII	*E. fredii*	GII	14±1.5	46.5±10.5	GII	5.12±4.57	39.87±25.061)	1073±118	2459±57
GE7W	GII	*E. fredii*	GII	42.3±6.4	66.7±2.5	GII	26.99±17.02)	31.81±11.2	1438.3±99*	2698.7±108
GE6W	GII	*E. fredii*	GII	46.6±102)	89.7±222)	GII	8.65±7.66	28.13±8.34	1939.3±138*2)	3177.7±399*1)
GE24W	GII	*E. fredii*	GII	41.6±3.7	105.3±161)	GII	27.04±3.491)	20.73±6.05	1307.7±223	2266.3±415
USDA110	—	*B. japonicum*	—	16.6±3.7	63.5±17.5	—	19.51±6.91	38.29±8.9	1370.3±98	2807±89*
Un-inoculated plant	—	—	—	0	0	—	0	0	1020.7±115	1940±94

**Part B:** Plant response to the inoculation groups of the *Bradyrhizobium* type in *Ensifer* background.										

KS2	GII	*E. fredii*	GIa	34.6±6.8	102.7±55	GIb	16.43±8.14	32.12±20.61	1651.7±156*	2933.3±479*

Un-inoculated plant	—	—	—	0	0	—	0	0	1020.7±115	1940±94

**Part C:** Plant response to the inoculation groups of the *Bradyrhizobium* type										

Isolate name	Phylogenetic groups of 16S rRNA	Related species	Phylogenetic groups of *nodD*	Nodule Numbers/plant (Stine3300)	Nodule Numbers/plant (Enrei)	Phylogenetic groups of *nifD*	ARA (μmol ethylene produced/hr/dry weight of nodule) (Stine3300)	ARA (μmol ethylene produced/hr/dry weight of nodule) (Enrei)	Biomass Stine 3300 (mg plant^−1^)	Biomass Enrei (mg plant^−1^)

PS2	GIa	*B. yuanmingense*	GIb	24.3±5.6	21.3±12	GIa	98.43±24.92)	29.4±11.2	1271.3±77*	1907±201
KS3	GIa	*B. yuanmingense*	GIb	53±26.31)	126±8.61)	GIa	44.97±12.3	51.77±8.5	1135.7±45*	2273±62*1)
PS8	GIc	*B. liaoningense*	GIb	23±7.2	9.6±2.5	GIa	73.86±25.1	51.9±26.3	1358.3±76*	2084±246
BgS4	GIb	unknown	GIa	32.7±7.6	56±5.2	GIb	83.83±39.4	97.17±20.9	1430.7±101*	2187.3±86*
PS3	GIb	unknown	GIa	27±8.6	54±5.5	GIb	92.26±32.7	62.86±28.6	1317.0±27*	2069±94
GE3	GIc	*B. liaoningense*	GIa	34.5±2.02)	92.3±222)	GIb	67.21±16.9	142.46±30.21)	1461.0±66*2)	2247±63*2)
KS12	GIc	*B. liaoningense*	GIa	12.3±3.5	53±28	GIb	109.58±30.31)	123.47±40.32)	690.3±164	2053.7±155
GE10	GIc	*B. liaoningense*	GIa	30.7±4.2	81.6±16	GIb	53.91±14.4	72.02±32.1	1667.3±109*1)	2130.3±249*
Un-inoculated plant	—	—	—	0	0	—	0	0	811.5±88	1645±56

* Value is significantly different from the control within each column in plant biomass production (*P*<0.05).

1) and 2) show the first and second values of each symbiotic performance of the tested isolates to soybeans.

**Table 3:** Phylogenetic analysis of indigenous soybean-root nodule rhizobia isolated from Afghanistan soils and used in two soybean cultivar trap hosts

Isolates	Sampling sites	Location	Bio-climatic zone	Soybean cultivar of the trap host	16S rRNA	Related species	*nodD1*	*nifD*	Accession numbers		

									16S rRNA	*nodD1*	*nifD*
BgS4	4	Baghlan	Semi-arid climate	Stine3300	GIb	Unknown	GIa	GIb	AB901354	LC009311	AB982207
BgS3	4	Baghlan	Semi-arid climate	Stine3300	GIc	*B. liaoningense*	GIa	GIb	AB901326	LC009306	AB982179
BgS2	4	Baghlan	Semi-arid climate	Stine3300	GIc	*B. liaoningense*	GIa	GIb	AB901325	LC009304	AB982178

KS11W	5	Kunduz	Semi-arid climate	Stine3300	GII	*E. fredii*	GII	GII	AB901322	LC009373	AB982175
KS10W	5	Kunduz	Semi-arid climate	Stine3300	GII	*E. fredii*	GII	GII	AB901324	LC009372	AB982177
KS2	5	Kunduz	Semi-arid climate	Stine3300	GII	*E. fredii*	GIa	GIb	AB901361	LC009324	AB982214
					
KS7	5	Kunduz	Semi-arid climate	Stine3300	GIa	*B. yuanmingense*	GIb	GIb	AB901362	LC009335	AB982215
KS3	5	Kunduz	Semi-arid climate	Stine3300	GIa	*B. yuanmingense*	GIb	GIa	AB901363	LC009337	AB982216
KS6	5	Kunduz	Semi-arid climate	Stine3300	GIb	Unknown	GIa	GIb	AB901356	LC009326	AB982209
KS10	5	Kunduz	Semi-arid climate	Stine3300	GIb	Unknown	GIa	GIb	AB901353	LC009322	AB982206
KS5	5	Kunduz	Semi-arid climate	Stine3300	GIb	Unknown	GIa	GIb	AB901355	LC009325	AB982208
KS11	5	Kunduz	Semi-arid climate	Stine3300	GIc	*B. liaoningense*	GIa	GIb	AB901352	LC009330	AB982205
KS12	5	Kunduz	Semi-arid climate	Stine3300	GIc	*B. liaoningense*	GIa	GIb	AB901331	LC009323	AB982184

GE11	1	Nangarhar	Hot desert climate	Enrei	GII	*E. fredii*	GII	GII	AB901294	LC009343	AB982147
GE12W	1	Nangarhar	Hot desert climate	Enrei	GII	*E. fredii*	GII	GII	AB901295	LC009344	AB982148
GE1W	1	Nangarhar	Hot desert climate	Enrei	GII	*E. fredii*	GII	GII	AB901296	LC009345	AB982149
GE20W	1	Nangarhar	Hot desert climate	Enrei	GII	*E. fredii*	GII	GII	AB901297	LC009346	AB982150
GE27W	1	Nangarhar	Hot desert climate	Enrei	GII	*E. fredii*	GII	GII	AB901298	LC009348	AB982151
GE2W	1	Nangarhar	Hot desert climate	Enrei	GII	*E. fredii*	GII	GII	AB901299	LC009349	AB982152
GE4W	1	Nangarhar	Hot desert climate	Enrei	GII	*E. fredii*	GII	GII	AB901300	LC009350	AB982153
GE5W	1	Nangarhar	Hot desert climate	Enrei	GII	*E. fredii*	GII	GII	AB901301	LC009351	AB982154
GE6W	1	Nangarhar	Hot desert climate	Enrei	GII	*E. fredii*	GII	GII	AB901302	LC009352	AB982155
GE7	1	Nangarhar	Hot desert climate	Enrei	GII	*E. fredii*	GII	GII	AB901303	LC009353	AB982156
GE8W	1	Nangarhar	Hot desert climate	Enrei	GII	*E. fredii*	GII	GII	AB901304	LC009354	AB982157
GE9	1	Nangarhar	Hot desert climate	Enrei	GII	*E. fredii*	GII	GII	AB901305	LC009355	AB982158
GE24W	1	Nangarhar	Hot desert climate	Enrei	GII	*E. fredii*	GII	GII	AB901323	LC009347	AB982176
					
GE10	1	Nangarhar	Hot desert climate	Enrei	GIc	*B. liaoningense*	GIa	GIb	AB901327	LC009312	AB982180
GE12	1	Nangarhar	Hot desert climate	Enrei	GIc	*B. liaoningense*	GIa	GIb	AB901328	LC009319	AB982181
GE13	1	Nangarhar	Hot desert climate	Enrei	GIc	*B. liaoningense*	GIa	GIb	AB901329	LC009308	AB982182
GE17	1	Nangarhar	Hot desert climate	Enrei	GIc	*B. liaoningense*	GIa	GIb	AB901332	LC009313	AB982185
GE18	1	Nangarhar	Hot desert climate	Enrei	GIc	*B. liaoningense*	GIa	GIb	AB901333	LC009315	AB982186
GE23	1	Nangarhar	Hot desert climate	Enrei	GIc	*B. liaoningense*	GIa	GIb	AB901335	LC009316	AB982188
GE25	1	Nangarhar	Hot desert climate	Enrei	GIc	*B. liaoningense*	GIa	GIb	AB901336	LC009317	AB982189
GE26	1	Nangarhar	Hot desert climate	Enrei	GIc	*B. liaoningense*	GIa	GIb	AB901337	LC009318	AB982190
GE28	1	Nangarhar	Hot desert climate	Enrei	GIc	*B. liaoningense*	GIa	GIb	AB901338	LC009310	AB982191
GE28b	1	Nangarhar	Hot desert climate	Enrei	GIc	*B. liaoningense*	GIa	GIb	AB901339	LC009307	AB982192
GE3	1	Nangarhar	Hot desert climate	Enrei	GIc	*B. liaoningense*	GIa	GIb	AB901340	LC009320	AB982193
GE16	1	Nangarhar	Hot desert climate	Enrei	GIc	*B. liaoningense*	GIa	GIb	AB901330	LC009314	AB982195
GE22	1	Nangarhar	Hot desert climate	Enrei	GIc	*B. liaoningense*	GIb	GIb	AB901334	LC009340	AB982187
				
GS1	1	Nangarhar	Hot desert climate	Stine3300	GII	*E. fredii*	GII	GII	AB901306	LC009356	AB982159
GS13W	1	Nangarhar	Hot desert climate	Stine3300	GII	*E. fredii*	GII	GII	AB901307	LC009357	AB982160
GS16b	1	Nangarhar	Hot desert climate	Stine3300	GII	*E. fredii*	GII	GII	AB901308	LC009358	AB982161
GS19b	1	Nangarhar	Hot desert climate	Stine3300	GII	*E. fredii*	GII	GII	AB901309	LC009359	AB982162
GS2	1	Nangarhar	Hot desert climate	Stine3300	GII	*E. fredii*	GII	GII	AB901310	LC009360	AB982163
GS21b	1	Nangarhar	Hot desert climate	Stine3300	GII	*E. fredii*	GII	GII	AB901311	LC009361	AB982164
GS23	1	Nangarhar	Hot desert climate	Stine3300	GII	*E. fredii*	GII	GII	AB901312	LC009362	AB982165
GS24	1	Nangarhar	Hot desert climate	Stine3300	GII	*E. fredii*	GII	GII	AB901313	LC009363	AB982166
GS25	1	Nangarhar	Hot desert climate	Stine3300	GII	*E. fredii*	GII	GII	AB901314	LC009364	AB982167
GS27	1	Nangarhar	Hot desert climate	Stine3300	GII	*E. fredii*	GII	GII	AB901315	LC009365	AB982168
GS28	1	Nangarhar	Hot desert climate	Stine3300	GII	*E. fredii*	GII	GII	AB901316	LC009366	AB982169
GS3	1	Nangarhar	Hot desert climate	Stine3300	GII	*E. fredii*	GII	GII	AB901317	LC009367	AB982170
GS4	1	Nangarhar	Hot desert climate	Stine3300	GII	*E. fredii*	GII	GII	AB901318	LC009368	AB982171
GS7	1	Nangarhar	Hot desert climate	Stine3300	GII	*E. fredii*	GII	GII	AB901319	LC009369	AB982172
GS8	1	Nangarhar	Hot desert climate	Stine3300	GII	*E. fredii*	GII	GII	AB901320	LC009370	AB982173
GS9W	1	Nangarhar	Hot desert climate	Stine3300	GII	*E. fredii*	GII	GII	AB901321	LC009371	AB982174
					
GS14	1	Nangarhar	Hot desert climate	Stine3300	GIc	*B. liaoningense*	GIa	GIb	AB901341	LC009309	AB982194
GS16	1	Nangarhar	Hot desert climate	Stine3300	GIc	*B. liaoningense*	GIa	GIb	AB901342	LC009336	AB982195
GS16C	1	Nangarhar	Hot desert climate	Stine3300	GIc	*B. liaoningense*	GIa	GIb	AB901343	LC009334	AB982196
GS19	1	Nangarhar	Hot desert climate	Stine3300	GIc	*B. liaoningense*	GIa	GIb	AB901344	LC009332	AB982197
GS20	1	Nangarhar	Hot desert climate	Stine3300	GIc	*B. liaoningense*	GIa	GIb	AB901345	LC009333	AB982198
GS21	1	Nangarhar	Hot desert climate	Stine3300	GIc	*B. liaoningense*	GIa	GIb	AB901346	LC009327	AB982199
GS22	1	Nangarhar	Hot desert climate	Stine3300	GIc	*B. liaoningense*	GIa	GIb	AB901347	LC009328	AB982200
GS6	1	Nangarhar	Hot desert climate	Stine3300	GIc	*B. liaoningense*	GIa	GIb	AB901349	LC009321	AB982202
GS9	1	Nangarhar	Hot desert climate	Stine3300	GIc	*B. liaoningense*	GIa	GIb	AB901350	LC009331	AB982203
GS5	1	Nangarhar	Hot desert climate	Stine3300	GIc	*B. liaoningense*	GIa	GIb	AB901348	LC009305	AB982201

PS2	3	Parwan	Cold semi-arid climate	Stine3300	GIa	*B. yuanmingense*	GIb	GIa	AB901358	LC009342	AB982211
PS6	3	Parwan	Cold semi-arid climate	Stine3300	GIa	*B. yuanmingense*	GIb	GIa	AB901360	LC009339	AB982213
PS5W	3	Parwan	Cold semi-arid climate	Stine3300	GIa	*B. yuanmingense*	GIb	GIa	AB901359	LC009341	AB982212
PS3	3	Parwan	Cold semi-arid climate	Stine3300	GIb	Unknown	GIa	GIb	AB901357	LC009329	AB982210
PS8	3	Parwan	Cold semi-arid climate	Stine3300	GIc	*B. liaoningense*	GIb	GIa	AB901351	LC009338	AB982204
