# Supplementary material for: Genetic Characterization of Soybean Rhizobia Isolated from Different Ecological Zones in North-Eastern Afghanistan
Source: Microbes Environ. 2017 Mar 17;32(1):71–9. doi: 10.1264/jsme2.ME16119 (PMC5371078; doi:10.1264/jsme2.ME16119)
Supplement: Supplementary file 1 [file 32_71_s1.pdf]

Table S1 Primers used in this study for the amplification and sequencing of 16S rRNA, *nodD1* and *nifD* genes

Primer	Sequence (5´-3´)	Reference
1F	AGTTTGATCCTGGCTC	(14)
3R	AAGGAGGTGATCCAGCC	
nodD1 134F	GCACCTATTTCCGCGATGAA	(31)
nodD1 880R	ATATCCGACGCATCCAGATG	
nifD 161F	TGCGRSGTRAAGTCSAAYAT	This study [(a,b,c)]
nifD 1435R	TCCATGTCKCGSGCGAARAT	

[a] *Bradyrhizobium japonicum* USDA 110 : Forward (145-164); Reverse (1399-1418)

[b] *Sinorhizobium fredii* HH103: Forward (160-179); Reverse (1408-1427)

[c] *Sinorhizobium meliloti* 1021: Forward (160-179); Reverse (1396-1415)
